# Supplementary material for: Consistent response of European summers to the latitudinal temperature gradient over the Holocene
Source: Nat Commun. 2025 Nov 19;16:9969. doi: 10.1038/s41467-025-65804-x (PMC12630748; doi:10.1038/s41467-025-65804-x)
Supplement: Supplementary file 1 — Supplementary Information [file 41467_2025_65804_MOESM1_ESM.pdf]

***Table-of-contents:***


1. The varved record of Diss Mere
2. The varved record of Nautajärvi
3. Compaction of recent varved sediments
4. Proxy calibration
5. Supplementary Figures and Tables

   Supplementary Figure 1

   Supplementary Figure 2

   Supplementary Figure 3

   Supplementary Figure 4

   Supplementary Table 1

   Supplementary Table 2

   Supplementary Table 3
6. References.

## 1. The varved record of Diss Mere

Diss Mere (52° 22'N, 1°6'E; 29 m a.s.l) is in East Anglia, UK and is a small, urban marl lake with a catchment area of 1.5 $km^2$. The current lake has no surface inflows or outflows and is 6 m deep with a smooth bathymetry[1]. Maximum depth is only reached in a small area in the middle of the lake, where four parallel sediment cores were obtained in September 2016 using a 90 mm diameter UWITEC piston corer (DISS16-A, B, C and D) (*25*). The cores were correlated using a total of 67 macroscopic visible and 129 microscopic marker layers and the best-preserved sections were combined to construct the continuous composite profile DISS-16 of 14.5 m length. Samples for petrographic thin sections (10 × 2 × 1 cm) were taken from the varves with 2 cm overlaps to enable continuous microfacies analysis, including correlation of marker layers. Thin sections were prepared according to a standard procedure including freeze-drying and impregnation with epoxy resin[2]. Detailed microfacies analysis, varve counting, and varve and sub-varve layer thickness measurements were performed on the petrographic thin sections using a Leica (M205C) stereo-zoom petrological microscope with plane- and cross-polarised light, at 80x. Varve counting and thickness measurements were performed for each seasonal layer along the ca 4.2 m long sequence of varved sediments. Lamina thickness measurements were replicated up to four times by three different researchers in some parts of the record[3].

The Holocene sediment record is 13 m long and goes back to 10,300 cal yr BP. The last 2 kyr (first 9 m of sediments) are made of massive to faintly-laminated sediments with an average sedimentation rate of ca. 0.5 cm/year, while the rest of the Holocene is recorded as a well-preserve varved sequence of 4.2 m of length with an average sedimentation rate (varve thickness) of 0.4 mm/ year. Sediments below are made of reworked diamicton (calcareous silt and sand with pebble-sized clasts)[3]. The loss of varve preservation and increase in sedimentation rate 2 kyr ago is due to human impact in the catchment[4]. It contributed to an increase in detrital input, sediment infilling and lake shallowing, influencing eventually to the semi-permanent anoxic state of the lake bottom[3], which is essential for varve preservation[5]. A 4-yr monitoring survey verifies that the lake bottom is anoxic for a few months only, however it demonstrates that monthly sedimentation today would reproduce the Holocene varve structure if the lamination would be preserved[1]. This allows testing the seasonality of the varve structure and sensitivity to seasonal weather.

The Diss Mere varves consist of a pale lamina made of authigenic calcite crystals, and a dark lamina composed of, primarily, crysophyceae cyst, planktonic centric diatoms, filaments of organic matter and micrite. Although three varve microfacies have been described along the record, all of these varve types represent variations of the couplet described above, which represent the 85% of the varves[3]. For example, we described a varve type with an extremely thin or absent calcite layer or another varve type including a monospecific diatom bloom. Based on the monitoring survey, we interpret it as interannual variability rather than changes in the lake system[1]. According to the phenologic characteristics of the Holocene varves described in previous studies, a thin layer made of cysts of the chrysophyceae and deposited at the top of the calcite laminae marks the end of the summer season[6], which suggest that the pale-calcite laminae is deposited during the summer and the dark-organic layer during the autumn/winter season. The seasonality of the varve is supported by the monitoring survey showing continuous accumulation of authigenic calcite in sediment traps from March to September coinciding with the period of lake stratification rather than heat extremes. Organic aquatic matter deposition occurs

from October to March with diatom blooms happening in October when the lake turnover starts and occasionally in February coinciding with episodes of strong winds[1]. Diatoms bloom are not preserved as single layers but included in the gyttja layer. The region in which Diss Mere is located has a temperate maritime climate. Annual mean temperature is 10.4 °C with maxima recorded in July and August and minima in February. Rainfall is evenly distributed throughout the year with an annual average precipitation of 627 mm. Wind speed is in inverse relation to temperature and has its maximum in February and minimum in July. The timing and duration of the lake seasons (i.e. stratification and mixing) match the annual cycle of temperature in the region, and agree with the timing and duration of the winter and summer weather seasons defined for western Europe[7]. In summary, summer-calcite layers are deposited from late spring to early autumn when the lake is stratified and calcite accumulation respond to summer temperatures; winter-organic layers are deposited from early autumn to late spring when the lake is mixed and changes in the accumulation of organic matter likely respond to winter storminess[1].

Holocene varves are well preserved except for a 10 cm interval between 12.04 m and 12.14 m of sediment depth (ca. 6.9 to 7.2 ka BP) where varves were interpolated. The varved sequence is, however, linked to a robust chronology that integrates varve counts (8473 varves), five radiocarbon dates, and two known tephra horizons, the Glen Garry and the OMH-185, into an Oxcal v4.4 Bayesian age-depth depositional model with a maximum absolute uncertainty of ± 55 years (95% confidence) at the bottom of the sequence[3,8,9].

## 2. The varved record of Nautajärvi

Lake Nautajärvi (61°48'N, 24°41'E; 103.7 m a.s.l) is located in central southern Finland and is an oval-shaped rural lake with surface area of 0.17 km$^2$ and a catchment area of 10.65 km$^2$. The lake has a maximum depth of 20 m in a single deposition basin that is located in the NNW part of the lake[10]. It is currently supplied by three inflows from the north, northwest and northeast, and there is one southern outflow. Varves are confined to the deepest basin (>18.5 m), which covers ca. 13% of the lake surface area.

The Holocene record from this basin is 6.6 m long and covers uninterruptedly varved sediments back to 9852 cal yr BP[10,11]. The documentation of varve chronology and varve/laminae analyses of the Nautajärvi sequence is based on three overlapping sediment cores (A, C, AS4) taken with a heavy gravity piston core[12] and one surface sediment core (JNA1) taken with a wedge-shaped freeze core[13] . The cores were correlated with mineral magnetic parameters and by using a total of ca. 200 visually discernible marker horizons that were located at 30-50-year intervals throughout the 6.6 m long sediment profile. (i.e. thicker minerogenic layers)[10,11]. Subsamples for analyses were prepared in 10×2×1 cm sections and embedded using a modification of the water-acetone-epoxy-change method[14]. Detailed microfacies analysis, varve counting, and varve and lamina layer thickness measurements were performed using scanning electron microscope back-scattered electron images (SEM BSEI), X-ray radiographs, and digital line-scan image analysis. Varves were analysed in between the marker horizons 5-6 times using different media, and deviations between counts were registered to represent the margin of error associated within the varve chronology(4).

The OIVA open data service maintained by the Finnish environmental authorities contains sporadic limnological measurements from Lake Nautajärvi since 1997 CE. These data

indicate that Lake Nautajärvi has seasonal water stratification and anoxia (<1 mg $l^{-1}$) near the bottom, indicating a dimictic nature supporting varve preservation. A 2.5-yr monitoring and sediment trapping survey verifies the dimictic nature of the lake and demonstrates the seasonal formation of clastic-biogenic varves[5,15]. The results also indicate that seasonal sediment fluxes in Lake Nautajärvi are controlled by climate parameters[15].

The present-day climate is continental, with a mean precipitation of 500–700 mm $yr^{-1}$, of which approximately one third falls as snow. The annual mean temperature is about +3 °C, the warmest month is July (+15 to +18 °C) and the coldest is January or February (-5 to -12 °C). Lake Nautajärvi is typically ice-covered for a period of 4 to 5 months, usually from December to April. Strong seasonal contrasts with winter snow and ice cover, spring flooding, appearance of easily erodible fine-grained clastic material in the catchment, and summer and mid-winter stratification of the water column (bottom anoxia) are the main causes for the deposition and preservation of clastic-biogenic varves in Lake Nautajärvi[15].

The basic structure of clastic-biogenic varves in the Lake Nautajärvi sequence is very simple[15]. Each varve consists of two layers, a pale clastic layer of detrital origin and a dark biogenic layer of particulate to fine-grained organic matter. The deposition and thickness of clastic layer is linked to snowmelt and spring discharge intensity, which depend on the severity and snow storage during the winter. In principle, thicker clastic laminae indicate an increase in spring runoff and erosion, which is interpreted to reflect low temperatures with uninterrupted snow accumulation during the winter season[15,16]. Hence, clastic particle accumulation provides a long-term record of variability in past winter weather[17,18]. Thin layers of clastic particles indicate mild and moist winters in Fennoscandia. Low mineral matter accumulation of clastic-biogenic varves to mild winters with a short ice cover period with minor snow accumulation interrupted by winter thawing periods[16,19]. Biogenic layer, on the other hand, forms a growing season component of clastic-biogenic varves. These laminae grade upwards from a more particulate summer organic matter to a finer-grained winter organic matter that settles to the lake bottom during winter ice cover time(3). In principle, biological primary production (autochthonous) is the major source of organic matter in Fennoscandian lakes with clastic-biogenic varves[15,20], where the organic production and accumulation rates of diatoms and chrysophyte cysts are more dependent on open-water seasonal processes (e.g. summer temperature, spring and autumnal overturn) than on rapid and short-lived episodes such as the spring discharge[21]. Annual precipitation also plays an important role in organic matter accumulation, because greater precipitation enhances organic matter and nutrient transport from the catchment, which both favours increased organic laminae thickness[20,21]. In summary, the winter-clastic layer is deposited in early spring but the amount of material deposited depends on the duration and severity of the winter season; the summer-organic layer is deposited when the lake is not cover by snow from spring to early autumn responding to temperature and precipitation in the summer (snow-free) season.

Nautajärvi varves are interruptedly preserved throughout the entire Holocene. The chronology of the sequence is based on multiple varve counts from a number of sediments cores[22], $^{137}$Cs dating of the uppermost section, and palaeomagnetic dating and comparison with other independently dated paleomagnetic secular variation (PSV) records in Finland and Sweden[16]. The varve counting is based on X-ray radiographs of epoxy embedded sediment samples and semi-automated digital image analysis[21]. The cumulative calculation errors for the entire sequence covering 9898 varves (for sediment cores taken in 1997-1998

CE) down to the sediment depth of 6.6 m was estimated to +83 (0.84%) and -97 (0.98%) varve years on the basis of replicated varve analyses. Combined with other means of dating, the chronological error is 1% down to 9852 cal yr BP[16]. New sediment cores have been taken in 2005, 2011 and 2023 CE, and the varve chronology is currently being updated to present day.

## 3. Compaction of recent varved sediments

Early diagenetic processes, i.e., the combination of physical, chemical and biological processes that occur in surface sediments following deposition, change lake sediment properties over time in several ways[27]. One of the main changes is sediment compaction, which is a function of reduced porosity and loss of water caused by the loading of later deposited material and a loss of labile sediment fractions, e.g., organic material[28]. Sediment compaction is particularly relevant to environmental and climate studies based on varved sediments, especially if varve thicknesses of recent varves are calibrated with instrumental data as sediment compaction is mostly affecting the uppermost part of the record[29]

To address this issue and remove the influence of sediment compaction in our palaeoclimate study, we have detrended the varve thickness data of the two varved records. Detrending the data also allow us to calibrate the high-frequency (annual to decadal) signal recorded in the sediments with the NOAA20-CR reanalysis summer weather days and to apply this calibration to the rest of the record.

## 4. Proxy calibration

Lake Nautajärvi still forms varves today, enabling the calibration of proxy data with instrumental data. According to Ojala and Alenius (2005)[10], variability of the varve thickness and thickness of the seasonal layers during 1881-1993 CE significantly correlate with annual, winter and summer precitation and spring temperature (Table S1), suggesting a combined response to different variables.

In this study, we are interested in the summer to winter ratio in order to reconstruct the European seasonal clock, and we introduce a new varve proxy that consists in the percentage of the annual signal represented by the summer and winter layers (i.e. % summer thickness and % winter thickness). For the proxy calibration, we use the original dataset of number of summer days created by Cassaou and Cattiaux (2016)[7], which is based computations from several combinations of data sets (EOBS high-resolution gridded product, NCEP and NOAA-20CR reanalyses). For this study we have used the NOAA-20CR dataset because is the longest one and overlaps with the proxy dataset.

Prior to the calibration, a detailed investigation of potential human impact influencing lake sedimentation in Nautajarvi during the calibration period covered by NOAA-20CR reanalysis data has been carried out. The land use history of the 20$^{th}$ century in the Nautajärvi catchment area can be traced from historical maps and aerial photographs (Maanmittaushallituksen uudistusarkisto 1914, 1925, National Land Survey of Finland 1958, 1977, 1986) (Figure S1). The earliest maps from 1910–1920 CE show two natural inflow streams, one from Ristijärvi (northwest) and the other from northeast, but also indicate cultivated fields upstream and along these stream systems. However, peatland areas in the Nautajärvi catchment were still untouched and only these two natural inlet

streams existed. The first ditches were excavated between 1920–1955 CE, which cover ca. 14% of current artificial streams network. These excavations focus on pristine peatlands 1-3 km northeast of Nautajärvi. In general, the forest drainage improved forest growth on pristine peatlands and the most intense ditching period in Finland was in the 1950s and 1960s. The forest peatland drainage has been reported to increase the sediment and nutrient load[23–25]. The nutrient load is reduced gradually and depends on the mire and catchment properties[26]. The increase in the discharge volume and materials is usually short-lived (few years) and forest drainage can also level the discharge with reducing peak runoffs and increase the runoff during low-flow periods[23,24]. The Nautajärvi catchment area experienced multiple ditching events between 1955–1975 CE, and one of these was an excavation of a new stream inlet into Nautajärvi from southeast (Figure S1). Ca. 65% of the artificial ditches in the Nautajärvi catchment area were excavated between 1955–1975 CE. Between years 1975–1985 CE no artificial ditches were excavated in the Nautajärvi catchment area and most of the fields were reforested due to termination of agricultural activities. After 1985 CE ditching activities related only to maintenance of existing drainage network.

The comparison and correlation of the proxy data with the reanalysis were conducted at annual resolution from 1900 to 2000 CE (Supplementary Fig. 2). During the period of intensified human impact due to ditching activities, the climate signal in the sediment decreases from $r = 0.76$ to $r = 0.41$(Supplementary Fig. 2). Although the number of datapoint is reduced to $n = 41$, we run the calibration from 1900 to 1940 CE only, when both, the summer and winter layers respond best to the duration of the climate seasons defined by Cassou and Cattiaux, 2016[7].

To assess potential temperature bias in this calibration (i.e., whether the proxy reflects only temperature seasonality), a similar analysis was conducted using local temperature measurements from meteorological stations near the lake. As anticipated by Ojala and Alenius (2005)[10] (Table S1), the correlation is weak ($r = 0.07$, $p = 0.53$, $n = 41$). This finding supports the interpretation that the seasonal variability recorded in the lake does not directly track local temperature, but instead reflects the combined influence of multiple meteorological factors shaping summer weather conditions.

Unfortunately, Diss Mere no longer preserves varves, preventing calibration. The palaeoenvironmental interpretation of the varves[3,4] together with the lake monitoring[1], support that the seasonal layers are a consequence of continue deposition during the lake seasons, instead of punctual events, and the duration of the lake seasons broadly coincide with the European climate seasons (not that lake monitoring data cover four year only preventing significant statistical correlations). Nevertheless, the Holocene evolution of the summer and winter recorded in both varved records is comparable, varying within the same range. This suggests that both sites capture the same variability and respond to the same driving factors. To perform the Holocene reconstruction of the number of summer days shown in the main text, we averaged the summer-to-annual ratio (%) from both records and applied the calibration derived from Nautajärvi (Figure S3).

## 5. Supplementary Figures and Tables

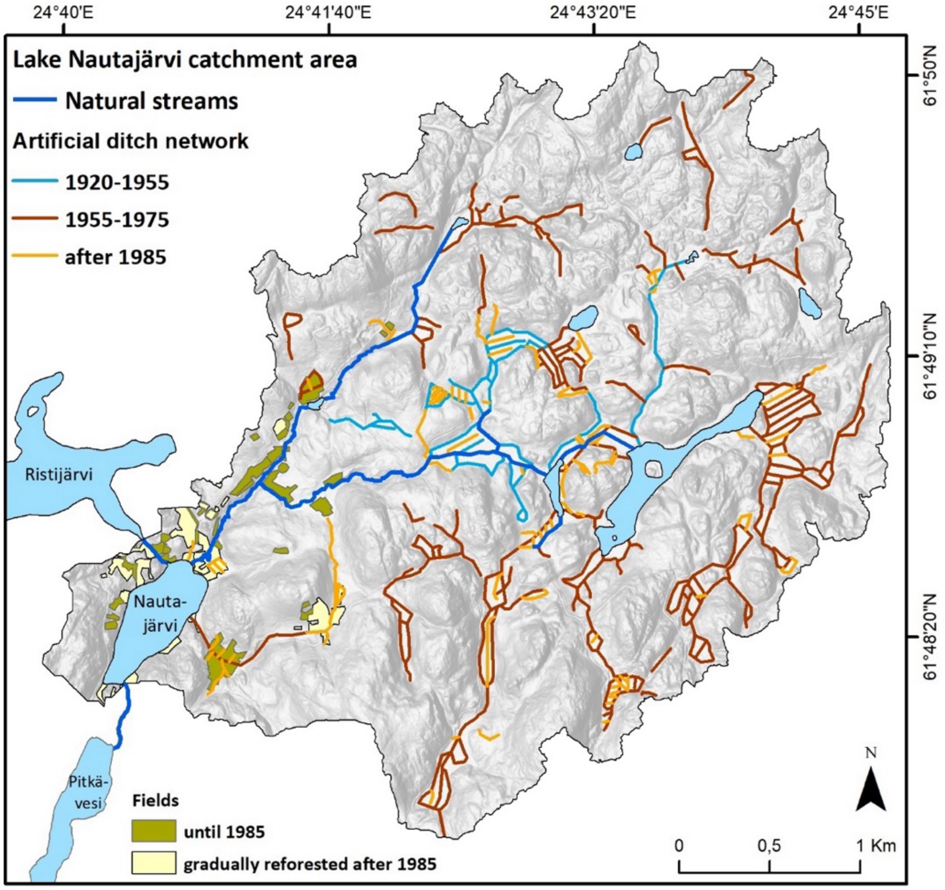


**Supplementary Figure 1.** Land-use history of the Lake Nautajärvi catchment area (1920–1985 CE). Field areas were delineated and categorized (fields and ditch network) based on historical maps (Maanmittaushallituksen uudistusarkisto 1914; NLS 1958, 1977, 1986 © National Land Survey of Finland, see open data resources below). The intervals and years shown in the legend correspond to the timing of the historical map surveys, not their publication years. The watershed area is based on Laser Scanning Data (0.5 p/m²) and Topographic Database (10/2024) © National Land Survey of Finland (CC BY 4.0). Open Data Resources © National Land Survey of Finland: Maanmittaushallituksen uudistusarkisto, 1914. MHA U Uudistuskartat ja -asiakirjat. H HÄMEEN LÄÄNI. Längelmäki. H46:2/21–40. Ristijärvi; N:o 1 Mattila, RN:o 1:1-4, halkominen (1914-1914). National Archives of Finland. https://astia.narc.fi/. ; Maanmittaushallituksen uudistusarkisto, 1925. Heikkilän perintötalon III osainen uudistus kartta (In finnish). Ristijärvi; N:o 2 Heikkilä, (1920-1921). The National Archives of Finland; NLS (National Land Survey of Finland, Maanmittaushallitus) 1958. Basic map 1:20 000, sheet 2142 12 Urtimojärvi. Maanmittaushallituksen kivipaino, Helsinki; NLS (National Land Survey of Finland, Maanmittaushallitus) 1977. Basic map 1:20 000, sheet 2142 12 Urtimojärvi. Maanmittaushallituksen karttapaino, Helsinki; NLS (National Land Survey of Finland, Maanmittaushallitus) 1986. Basic map 1:20 000, sheet 2142 12 Urtimojärvi. Maanmittaushallituksen karttapaino, Helsinki

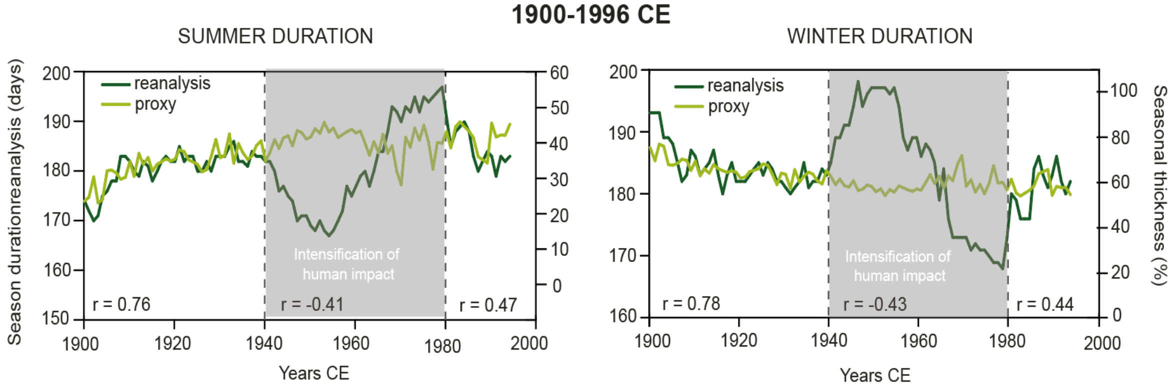


| Regression Statistics | |
|---|---|
| Multiple R | 0.75089038 |
| R Square | 0.56383637 |
| Adjusted R Square | 0.55265268 |
| Standard Error | 2.49383616 |
| Observations | 41 |

| Regression Statistics | |
|---|---|
| Multiple R | 0.75135247 |
| R Square | 0.56453054 |
| Adjusted R Square | 0.55336466 |
| Standard Error | 2.31084741 |
| Observations | 41 |

**Supplementary Figure 2. Proxy data and reanalysis comparison.** Season-to-annual ratios calculated as the percentage of seasonal lamina thickness records contributing to the total varve thickness from lake Nautajärvi (light green): %summer thickness (left) and %winter thickness (right). The seasonal-to annual ratio is compared to the duration of the European climate seasons (dark green) defined by Cassou and Cattiaux (2016)[7] during the period (1900 – 1996 CE). Computation from NOAA-20CR reanalysis, data provided by the authors[1]. Data are shown at annual resolution. Pearson's correlation coefficients are shown for the sub-periods (1900-1940 CE; 1940-1980 CE; 1980-1996 CE). As discussed in section 1.2., the impact of the ditching and intensified agriculture around the lake influenced the climate signal recorded in the sediments from 1940-1980 CE. Correlation increases after 1980 CE. We carried out the proxy calibration using the 1900-1940 CE period only, which includes 41 data points and provides a significant correlation with p-values lower than 0.001. Below each plot, regression statistics of the calibration models (summer and winter) are shown.

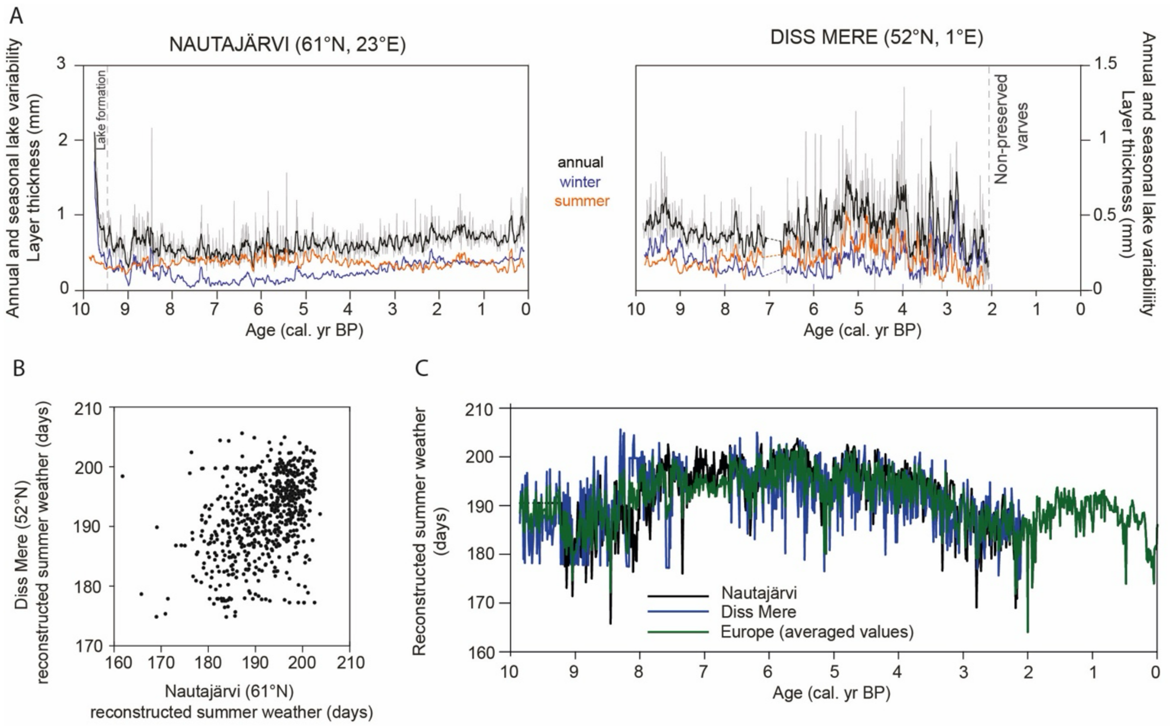


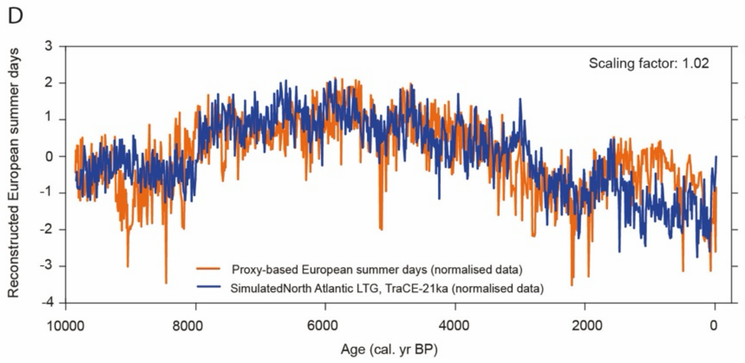


E

| | Summer days | LTG Anomaly (°C) |
|---|---|---|
| **Max** | 202 | 2.3 |
| **Min** | 164 | -2.3 |
| **Mean** | 191 | -0.33 |
| **Stdev** | 5.39 | 0.96 |

**Scaling factor: 5.61**

**r = - 0.6, p-value <0.001 (10-yr resolution)**
**r = - 0.8, p-value <0.001 (500-yr smooth)**

**Supplementary Figure 3.** (A) Holocene varve and lamina thickness records (raw data) of lake Nautajärvi, Finland (left) and Diss Mere, England (right). Annual data in grey and a 50-yr moving average of the varves (black), summer lamina (orange) winter lamina (blue). (B) Scatter plot of the relationship between the summer weather days reconstructed from the two proxy records. (C) Timeseries of the reconstructed summer days from Nautajärvi (back), Diss Mere (blue) and an averaged European signal (green), which is used in this study. (D) Holocene European summer days reconstruction (10-yr resolution) plotted against the simulated latitudinal temperature gradient (TraCE-21ka transient climate model) for the North Atlantic-European region (10-yr resolution). Data have been normalised for comparison and visualisation of the rate of change of the two timeseries. The ratio of the standard deviation between the two series (scaling factor) is 1.02, which support the proportionality between the two variables. (E) Timeseries (non-normalised) statistics. The scaling factor is calculated as the ratio of the standard deviations, i.e. Stdev (reconstruction) / Stdev (reanalysis).

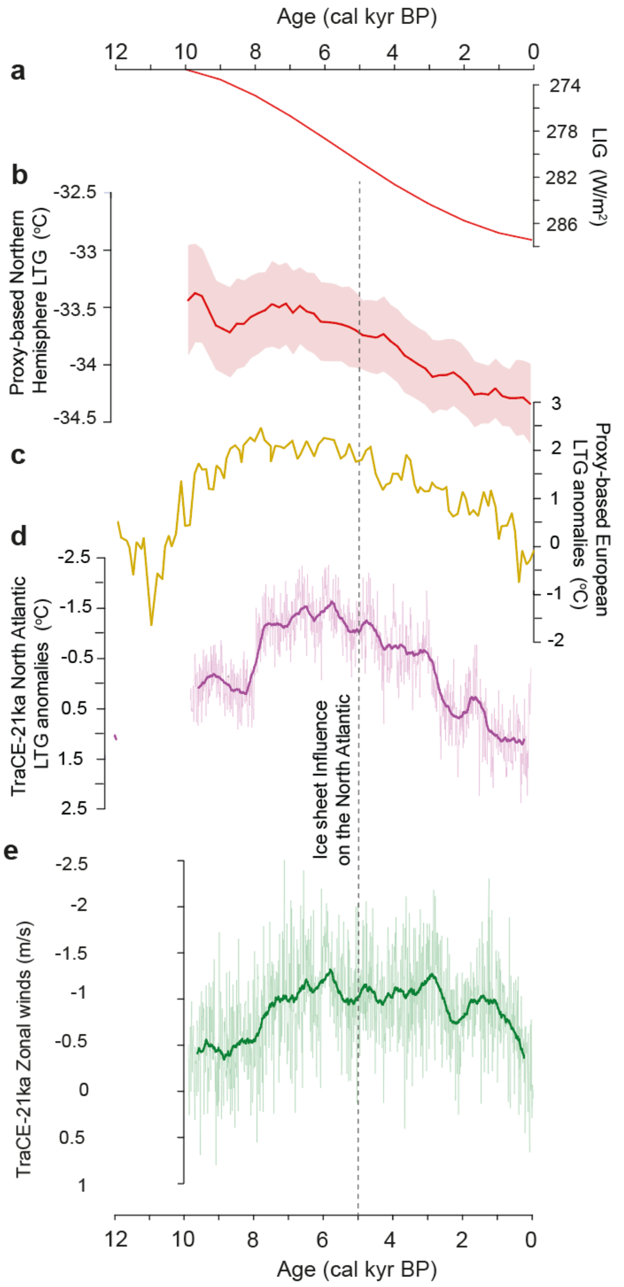


**Supplementary Figure 4. Climate drivers for seasonal weather persistence in the North Atlantic-European sector.** (a) Northern Hemisphere latitudinal insolation gradient (LIG)[30] calculated as the difference between low and high latitudes; (b) Proxy-based latitudinal temperature gradient (LTG) reconstruction for the Northern Hemisphere[31] calculated as the temperature difference between high and low latitudes. Note that data are plotted as in the original paper, so lower LTG values means stronger gradients as the gradient was calculated from high to low latitudes; (c) Proxy-based latitudinal temperature gradient reconstruction for Europe[32] calculated as the temperature difference between high and low latitudes. (d) TraCE-21ka North Atlantic LTG simulation (this study) calculated as the temperature difference between low and high latitudes; (e) and TraCE-21ka mid-latitude zonal wind speed simulation in the North Atlantic-European sector.

**Supplementary Table 1.** Pearson's correlation coefficients (r) for Lake Nautajärvi varve data and instrumental meteorological measurements of the Jyväskylä weather station between 1881-1993 CE. Modified from Ojala and Alenius (2005)[10]. * Marked correlations are significant at $p < 0.05$.

| Climate variable | Varve thickness | Winter layer | Summer layer |
|---|---|---|---|
| Annual Temp. | 0.187* | 0.153 | 0.189 |
| Winter Temp. | 0.129 | 0.078 | 0.139 |
| Spring Temp. | 0.331* | 0.305* | 0.324* |
| Summer Temp. | -0.130 | -0.099 | -0.134 |
| Annual Prec. | 0.420* | 0.398* | 0.408* |
| Winter Prec. | 0.367* | 0.300* | 0.372* |
| Spring Prec. | 0.124 | 0.162 | 0.107 |
| Summer Prec. | 0.320* | 0.339* | 0.299* |

**Supplementary Table *2*.** Summary of the dataset used and time period available for the instrumental data used in this study

| **Dataset** | **Temporal Coverage** | **Grid Size** | **References** |
|---|---|---|---|
| HadCRUT5 | 1850 - 2022 | 5° x 5° | Morice et al. (2021);<br>Osborn et al. (2021) |
| NASA GISTemp v4 | 1880 - 2022 | 2° x 2° | Lenssen et al (2019) |
| NOAA GlobalTemp | 1850 - 2022 | 5° x 5° | Zhang et al. (2019) |
| Berkeley Earth | 1850 - 2022 | 1° x 1° | Rohde and Hausfather (2020) |
| ERA5 | 1940 - 2022 | 0.5° x 0.5° | Hersbach et al (2020) |

**Supplementary Table 3.** Summary of the CMIP6 model simulations used in this study for the historical and SSP projection ensembles.

| Institution | Model |
|---|---|
| CSIRO (Australia) | ACCESS-CM2 |
| CSIRO (Australia) | ACCESS-ESM1-5 |
| Alfred Wegner institute, Helmholtz Centre for Polar and Marine Research (Germany) | AWI-CM-1-1-MR |
| Bejing Climate Centre (China) | BCC-CSM2-MR |
| Chinese Academy of Science (China) | CAS-ESM2-0 |
| Centro Euro-Mediterraneao sui Cambiamenti Climatici (Italy) | CMCC-CM2-SR5 |
| Centro Euro-Mediterraneao sui Cambiamenti Climatici (Italy) | CMCC-ESM2 |
| CNRM-CERFACS (France) | CNRM-CM46-1 |
| CNRM-CERFACS (France) | CNRM-CM6-1-HR |
| CNRM-CERFACS (France) | CNRM-ESM2-1 |
| Canadian Centre for Climate Modelling and Analysis (Canada) | CanESM5 |
| Canadian Centre for Climate Modelling and Analysis (Canada) | CanESM-Can0E |
| EC-Earth Consortium | EC-Earth3-Veg |
| EC-Earth Consortium | EC-Earth-Veg-LR |
| Institute of Physics (China) | FGOALS-g3 |
| National Oceanic and Atmospheric Administration (USA) | GFDL-ESM4 |
| NASA Goddard Institute for Space Studies (USA) | GISS-E2-1-G |
| Russian Academy of Science (Russia) | INM-CM4-8 |
| Russian Academy of Science (Russia) | INM-CM5-0 |
| Institut Pierre-Simon Laplace (France) | IPSL-CM6A-LR |
| JAMSTEC, NIES, AOTI, U. of Tokyo (Japan) | MIROC6 |
| Max Planck institute for Meteorology (Germany), and Deutsches Klimarechenzentrum (Germany) and Deutscher Wetterdienst (Germany) | MPI-ESM1-2-HR |
| Max Planck institute for Meteorology (Germany), and Deutsches Klimarechenzentrum (Germany) and Deutscher Wetterdienst (Germany) | MPI-ESM1-2-LR |

## 6. References


1. Boyall, L., Valcárcel, J. I., Harding, P., Hernández, A. & Martin-Puertas, C. Disentangling the environmental signals recorded in Holocene calcite varves based on modern lake observations and annual sedimentary processes in Diss Mere, England. *J Paleolimnol* **70**, 39–56 (2023).
2. Brauer, A. & Casanova, J. Chronology and depositional processes of the laminated sediment record from Lac d'Annecy, French Alps. *Journal of Paleolimnology* **25**, 163–177 (2001).
3. Martin-Puertas, C. *et al.* The first Holocene varve chronology for the UK: Based on the integration of varve counting, radiocarbon dating and tephrostratigraphy from Diss Mere (UK). *Quaternary Geochronology* **61**, 101134 (2021).
4. Peglar, S. M., Fritz, S. C. & Birks, H. J. B. Vegetation and Land-Use History at Diss, Norfolk, U.K. *The Journal of Ecology* **77**, 203 (1989).
5. Zolitschka, B., Francus, P., Ojala, A. E. K. & Schimmelmann, A. Varves in lake sediments – a review. *Quaternary Science Reviews* **117**, 1–41 (2015).
6. Peglar, S. M., Fritz, S. C., Alapieti, T., Saarnisto, M. & Birks, H. J. B. Composition and formation of laminated sediments in Diss Mere, Norfolk, England. *Boreas* **13**, 13–28 (1984).
7. Cassou, C. & Cattiaux, J. Disruption of the European climate seasonal clock in a warming world. *Nature Clim Change* **6**, 589–594 (2016).
8. Walsh, A. A., Blockley, S. P. E., Milner, A. M., Matthews, I. P. & Martin-Puertas, C. Complexities in European Holocene cryptotephra dispersal revealed in the annually

laminated lake record of Diss Mere, East Anglia. *Quaternary Geochronology* **66**, 101213 (2021).

9. Walsh, A. A., Blockley, S. P. E., Milner, A. M. & Martin-Puertas, C. Updated age constraints on key tephra markers for NW Europe based on a high-precision varve lake chronology. *Quaternary Science Reviews* **300**, 107897 (2023).
10. Ojala, A. E. K. & Alenius, T. 10000 years of interannual sedimentation recorded in the Lake Nautajärvi (Finland) clastic–organic varves. *Palaeogeography, Palaeoclimatology, Palaeoecology* **219**, 285–302 (2005).
11. Ojala, A. E. K. & Tiljander, M. Testing the fidelity of sediment chronology: comparison of varve and paleomagnetic results from Holocene lake sediments from central Finland. *Quaternary Science Reviews* **22**, 1787–1803 (2003).
12. Putkinen: Kullenbergin näytteenottimen uusi kevennetty... - Google Scholar. https://scholar.google.com/scholar_lookup?title=Kullenbergin+n%C3%A4ytteenottimen+uusi+kevennetty+malli+%28A+lighter+model+of+Kullenberg%27s+sampler%29&publication_year=1998&journal=Geologi&pages=22-23.
13. Renberg, I. Improved methods for sampling, photographing and varve-counting of varved lake sediments. *Boreas* **10**, 255–258 (1981).
14. Lamoureux, S. F. Embedding unfrozen lake sediments for thin section preparation. *J Paleolimnol* **10**, 141–146 (1994).
15. Ojala, A. E. K., Kosonen, E., Weckström, J., Korkonen, S. & Korhola, A. Seasonal formation of clastic-biogenic varves: the potential for palaeoenvironmental interpretations. *GFF* **135**, 237–247 (2013).

16. Haltia-Hovi, E., Saarinen, T. & Kukkonen, M. A 2000-year record of solar forcing on varved lake sediment in eastern Finland. *Quaternary Science Reviews* **26**, 678–689 (2007).

17. Ojala, A. E. K., Heinsalu, A., Saarnisto, M. & Tiljander, M. Annually laminated sediments date the drainage of the Ancylus Lake and early Holocene shoreline displacement in central Finland. *Quaternary International* **130**, 63–73 (2005).

18. Petterson, G., Renberg, I., Sjöstedt-de Luna, S., Arnqvist, P. & Anderson, N. J. Climatic influence on the inter-annual variability of late-Holocene minerogenic sediment supply in a boreal forest catchment. *Earth Surface Processes and Landforms* **35**, 390–398 (2010).

19. Tiljander, M., Saarnisto, M., Ojala, A. E. K. & Saarinen, T. A 3000-year palaeoenvironmental record from annually laminated sediment of Lake Korttajarvi, central Finland. *Boreas* **32**, 566–577 (2003).

20. Saarni, S., Saarinen, T. & Lensu, A. Organic lacustrine sediment varves as indicators of past precipitation changes: a 3,000-year climate record from Central Finland. *J Paleolimnol* **53**, 401–413 (2015).

21. De Stasio Jr., B. T., Hill, D. K., Kleinhans, J. M., Nibbelink, N. P. & Magnuson, J. J. Potential effects of global climate change on small north-temperate lakes: Physics, fish, and plankton. *Limnology and Oceanography* **41**, 1136–1149 (1996).

22. Ojala, A. E. K. & Saarinen, T. Palaeosecular variation of the Earth's magnetic field during the last 10000 years based on the annually laminated sediment of Lake Nautäjarvi, central Finland. *The Holocene* **12**, 391–400 (2002).

23. Seuna, P. Influence of forestry draining on runoff and sediment discharge in the Ylijoki basin, North Finland. *Aqua Fennica* 3–16 (1982).

24. Sandman, O., Lichu, A. & Simola, H. Drainage ditch erosion history as recorded in the varved sediment of a small lake in East Finland. *J Paleolimnol* **3**, 161–169 (1990).

25. Ahtiainen, M. & Huttunen, P. Long-term effects of forestry managements on water quality and loading in brooks. *Boreal Environment Research* (1999).

26. Sarkkola, S. KUSTAA -työkalu valuma-alueen vesistökuormituksen laskentaan. (2014).

27. Berner: Early diagenesis: a theoretical approach - Google Scholar. https://scholar.google.com/scholar_lookup?hl=en&publication_year=1980&author=R.A.+Berner&title=Early+Diagenesis%3A+A+Theoretical+Approach.

28. Gälman, V., Petterson, G. & Renberg, I. A Comparison of Sediment Varves (1950–2003 AD) in Two Adjacent Lakes in Northern Sweden. *J Paleolimnol* **35**, 837–853 (2006).

29. Maier, D. B., Rydberg, J., Bigler, C. & Renberg, I. Compaction of recent varved lake sediments. *GFF* **135**, 231–236 (2013).

30. Laskar, J. *et al.* A long-term numerical solution for the insolation quantities of the Earth. *A&A* **428**, 261–285 (2004).

31. Routson, C. C. *et al.* Mid-latitude net precipitation decreased with Arctic warming during the Holocene. *Nature* **568**, 83–87 (2019).

32. Davis, B. A. S. & Brewer, S. Orbital forcing and role of the latitudinal insolation/temperature gradient. *Clim Dyn* **32**, 143–165 (2009).
