# Supplementary material for: Consistent response of European summers to the latitudinal temperature gradient over the Holocene
Source: Nat Commun. 2025 Nov 19;16:9969. doi: 10.1038/s41467-025-65804-x (PMC12630748; doi:10.1038/s41467-025-65804-x)
Supplement: Supplementary file 2 — Description of Additional Supplementary Files [file 41467_2025_65804_MOESM2_ESM.pdf]

Description of Additional Supplementary Files

File name: Supplementary Data 1

Description: Data for Figure 2. Regression model results between the reanalysis number of summer days (NOAA20-CR) and the summer-to-annual ratio expressed as percentage. Columns A-D are data of the proxy calibration used in Figure 2 a-b. Column E-N are data of the Holocene reconstruction of number of summer days from the varved records of Nautajärvi and Diss Mere plotted in Figure 2c, and Figure S3.

File name: Supplementary Data 2

Description: European summer days reconstruction and uncertainties. Data plotted in Figure 3.
